# Hospitalists Are Already Using AI—Why Implementation Will Determine Its Impact

**DOI:** 10.2196/97419

**Published:** 2026-06-01

**Authors:** Anna Maw, Aakriti Pandita, Marisha Burden

**Affiliations:** 1 Division of Hospital Medicine, University of Colorado School of Medicine, Leprino Building, 4th Floor, Mailstop F-782, 12401 E 17th Avenue, Aurora, CO, 80045, United States, 1 720 848 4289

**Keywords:** artificial intelligence, generative AI, hospital medicine, clinical decision support systems, health care technology adoption, implementation science

## Abstract

The adoption of artificial intelligence (AI) into clinical practice is accelerating, outpacing the development of organizational guidance, training, and governance. A recent study indicated that two-thirds of hospitalists are using AI, particularly large language model (LLM)–based platforms, in their clinical work. However, as with prior disruptive health technologies, adoption alone does not ensure meaningful improvement in care. Drawing on lessons from electronic health record implementation, we argue that AI’s ultimate impact will be determined not by use rates, but by implementation quality and fit. Poorly implemented digital tools have been shown to increase clinician workload and burnout, despite their intended benefits. Early evidence on LLM-based diagnostic AI further underscores this risk: clinical-decision making supported by AI may be suboptimal when integration, training, and workflow design are inadequate. To provide value, AI tools must be thoughtfully embedded into clinical reasoning processes through evidence-informed training, intentional workflow design, and supportive organizational culture. As AI technologies are rapidly adopted, three priorities come into focus: training clinicians on AI inputs and interpreting outputs, applying implementation science frameworks for AI deployment in clinical environments, and establishing strategies for ongoing evaluation of the impact of AI tools over time. Implementation science frameworks offer practical guidance to assess workflow integration, training needs, infrastructure, and potential unintended consequences that can then inform adaptation of implementation strategies to enhance contextual fit. In parallel, learning health system infrastructure can enable continuous monitoring and iterative adaptation using routinely collected clinical and workflow data that reflect the value of the intervention across the quintuple aim of clinical outcomes, health equity, cost, and patient and clinician experience. AI adoption in hospital medicine is likely inevitable. Its ability to advance the quintuple aim will depend on how effectively these tools are implemented, supported, evaluated, and adapted in practice.

The integration of artificial intelligence (AI) into clinical practice is accelerating, with clinicians adopting AI tools faster than health systems can guide and govern their use, often without structured training or guidance to interpret outputs or manage potential risk. In a recent study by Bagla and colleagues [1], two-thirds of hospitalists reported using AI platforms in clinical practice, most commonly large language model (LLM)–based tools, and were doing so largely in the absence of health system integration. Hospitalists reported using these tools primarily to support clinical decision-making, including generating differential diagnoses and management options, with a strong preference for medical-specific platforms over general-purpose applications. Yet as with any disruptive health technology, adoption alone should not be considered success. Prior experience has shown that widespread uptake of technology does not guarantee progress toward the quintuple aim of optimizing patient outcomes, health equity, cost, and patient and clinician experience [2]. The work by Bagla et al [1] provides an important foundation by highlighting how AI is already being used in practice, with broader applications and tools likely to continue expanding. The critical next question is whether and under what conditions these patterns of use translate into meaningful improvements across the quintuple aim, particularly as health systems begin to consider how these tools should be implemented, supported, and governed.

Whether clinician-initiated (as with many current AI tools) or system-led (as with electronic health record [EHR] implementation), health technologies often fall short when implementation, training, and workflow redesign lag behind adoption. The history of EHR implementation provides a cautionary example of adopting technology without careful attention to implementation and contextual fit. While EHRs have contributed to important gains in standardization and safety, they have also introduced risks, including interoperability challenges, clinician inefficiencies and cognitive burden, and even downstream patient safety concerns [3]. Importantly, these harms largely reflect design and implementation failures rather than what the technology itself can offer. As emerging AI technologies are rapidly adopted, three priorities come into focus: training clinicians on prompt engineering and interpreting outputs, applying implementation science frameworks for AI deployment in clinical environments, and establishing strategies for ongoing evaluation of the impact of AI tools over time.

As health systems move toward integration of various AI technologies (including LLM-based generative AI solutions and beyond), emerging evidence from randomized controlled trials highlights how implementation and training critically shape their impact. For example, when comparing diagnostic performance of LLMs across three groups—clinicians alone, clinicians with AI assistance, and AI alone—Goh and colleagues [4] reported that AI assistance did not improve diagnostic reasoning of clinicians and AI alone outperformed clinicians. This does not necessarily imply that clinicians should be replaced; rather, it reflects a critical gap in implementation. Simply adding AI to clinical practice is insufficient. Without evidence-informed training and structured approaches to use, clinicians may fail to appropriately prompt, interpret, contextualize, and act on AI outputs, limiting the clinical benefits that could be achieved. Additional evidence from randomized controlled trials further demonstrates that it is not only *whether* AI is used, but *how* and *when* it is integrated into clinical reasoning that shapes outcomes. Everett et al [5] demonstrated that when clinicians received targeted training and used intentionally designed AI-first or AI-second workflows, diagnostic accuracy improved markedly, highlighting that *how* AI is integrated influences outcomes. Similarly, early studies of AI-enabled documentation tools, such as AI scribes, show promise but also demonstrate variable benefit across care settings and workflows [6].

To advance the quintuple aim, AI use in clinical care must be approached as more than simply deploying a new tool, with careful attention to how it is introduced, supported, and used in practice [7]. This is particularly important for LLM-based platforms being used to support clinical decision-making, whose outputs vary by prompt input and can include overly confident, sometimes erroneous output that could negatively impact clinician decision-making. Implementation science frameworks provide practical scaffolding for operational decision-making, particularly as AI tools are rapidly deployed into clinical environments. For instance, the pragmatic robust implementation and sustainability model (PRISM) can help health system leaders systematically assess key factors, such as workflow integration, organizational culture, infrastructure (such as training), and external pressures, and anticipate and measure how these elements influence outcomes and unintended consequences [8]. In practice, this means moving beyond “Does it work?” to more actionable questions: “Where in our workflows should this tool be used?” “Who needs training?” “What could go wrong?” “How will we monitor and improve performance over time?” and “What are the impacts on patients, the workforce, and the health system?” At the same time, responsible use extends beyond organizational strategy to individual clinicians who are already incorporating AI tools into their practice. Clinicians must understand the strengths and limitations of the AI tools they use clinically, and how the outputs of these tools influence their decision-making. Clinicians must also ensure that their tool use aligns with Health Insurance Portability and Accountability Act (HIPAA) requirements and organizational policies.

Finally, to understand whether training and deployment strategies are working in practice, implementation of AI tools at scale will require ongoing health system evaluation and adaptation rather than one-time deployment. Learning health system infrastructure, which allows system leaders to leverage data collected through routine clinical care, can enable continuous, near-real-time monitoring of process and outcome measures, making timely iterative improvement feasible at scale [9]. Fortunately, much of these data already exist within the EHR, from audit logs that reveal workflow patterns to clinical data that track patient outcomes [10]. However, measurement must extend beyond what is easily captured: periodic assessments of clinician experience and workflow burden are critical, as are economic evaluations to ensure that AI tools deliver on their investment.

Ultimately, AI adoption in hospital medicine (and beyond) will only continue, but its impact on critical outcomes remains uncertain. The central challenge is no longer whether these tools are adopted but whether they are implemented in ways that meaningfully improve outcomes for patients, clinicians, and health systems.

## Abbreviations

AI: artificial intelligence
EHR: electronic health record
HIPAA: Health Insurance Portability and Accountability Act
LLM: large language model
PRISM: pragmatic robust implementation and sustainability model

## References

[1] Bagla P, Hanna J, Marthambadi B, Watkins S (2026). Patterns of AI use in clinical work by hospitalists: survey study. J Med Internet Res.

[2] Nundy S, Cooper LA, Mate KS (2022). The quintuple aim for health care improvement: a new imperative to advance health equity. JAMA.

[3] Shanafelt TD, Dyrbye LN, Sinsky C (2016). Relationship between clerical burden and characteristics of the electronic environment with physician burnout and professional satisfaction. Mayo Clin Proc.

[4] Goh E, Gallo R, Hom J (2024). Large language model influence on diagnostic reasoning: a randomized clinical trial. JAMA Netw Open.

[5] Everett SS, Bunning BJ, Jain P (2026). From tool to teammate in a randomized controlled trial of clinician-AI collaborative workflows for diagnosis. NPJ Digit Med.

[6] Rotenstein LS, Holmgren AJ, Thombley R (2026). Changes in clinician time expenditure and visit quantity with adoption of artificial intelligence-powered scribes: a multisite study. JAMA.

[7] Skivington K, Matthews L, Simpson SA (2021). A new framework for developing and evaluating complex interventions: update of Medical Research Council guidance. BMJ.

[8] Glasgow RE, Trinkley KE, Ford B, Rabin BA (2024). The application and evolution of the practical, robust implementation and sustainability model (PRISM): history and Innovations. Glob Implement Res Appl.

[9] Maw AM, Trinkley KE, Glasgow RE (2024). The role of pragmatic implementation science methods in achieving equitable and effective use of artificial intelligence in healthcare. J Gen Intern Med.

[10] Burden M, Dyrbye L (2025). Evidence-based work design - bridging the divide. N Engl J Med.

